# Ictal Fear or Panic Attack, This Is the Question—A Video–EEG Study

**DOI:** 10.3390/brainsci14060594

**Published:** 2024-06-12

**Authors:** Francesco Castellana, Grazia D’Onofrio, Filomena Ciccone, Maria Teresa Di Claudio, Maura Pugliatti, Teresa Popolizio, Giuseppe d’Orsi

**Affiliations:** 1 Neurology Unit, Epilepsy Center, IRCCS Casa Sollievo della Sofferenza, San Giovanni Rotondo, 71013 Foggia, Italy; francesco.castellana@edu.unife.it (F.C.); mariateresadiclaudio@gmail.com (M.T.D.C.); g.dorsi@operapadrepio.it (G.d.); 2 Unit of Clinical Neurology, Department of Neuroscience and Rehabilitation, University of Ferrara, 44121 Ferrara, Italy; maura.pugliatti@unife.it; 3 Clinical Psychology Service, Health Department, Fondazione IRCCS Casa Sollievo della Sofferenza, San Giovanni Rotondo, 71013 Foggia, Italy; f.ciccone@operapadrepio.it; 4 Complex Unit of Radiology, Fondazione IRCCS Casa Sollievo della Sofferenza, San Giovanni Rotondo, 71013 Foggia, Italy; t.popolizio@operapadrepio.it

**Keywords:** ictal fear, panic attack, video–EEG, temporal lobe epilepsy

## Abstract

Panic disorder (PD) and focal epilepsy, in particular, temporal lobe epilepsy, often present diagnostic challenges due to overlapping clinical manifestations. This article describes the case of a 25-year-old female, misdiagnosed with PD for 15 years, whose recurring episodes of sudden fear, palpitations, and nausea were later identified as manifestations of focal epilepsy. Initially unresponsive to conventional anti-anxiety medications, the patient’s correct diagnosis was only established through comprehensive electro-clinical, neuropsychological, and neuroimaging evaluations during her admission to our research hospital. Long-term video–EEG monitoring (LTVEM) played a pivotal role in identifying the epileptic nature of her episodes, which were characterized by paroxysmal activity in the right temporal and zygomatic regions, consistent with the location of a dysplastic lesion in the right amygdala, as revealed by high-resolution magnetic resonance imaging. These findings underline the importance of considering focal epilepsy in the differential diagnosis of PD, especially in cases refractory to standard psychiatric treatments. The misdiagnosis of epilepsy as PD can lead to significant delays in appropriate treatment, potentially exacerbating the patient’s condition and affecting their quality of life. This case emphasizes the necessity of a multidisciplinary approach and the utilization of advanced diagnostic tools like LTVEM in elucidating the underlying causes of paroxysmal psychiatric symptoms.

## 1. Introduction

Panic disorder (PD) is one of the most common psychiatric disorders, with a 12-month prevalence rate of 1.8 percent according to a systematic review of studies conducted among European cohorts [1]. PD is delineated by recurrent and unforeseen panic attacks (PAs), coupled with a persistent apprehension of experiencing another attack or of the consequences that another attack might have, or with a behavioral change related to the attacks that endures at least for one month [2]. Fear is an affective symptom frequently linked to idiopathic PAs, but it also emerges as an ictal symptom in a notable subset of patients, ranging from 10 to 15%, who exhibit temporal lobe epilepsy [3]. It is worth noting that the diagnosis of focal seizures in patients with temporal lobe epilepsy often encounters delays due to the potential misdiagnosis of these seizures as psychiatric disturbances, especially when fear assumes a prominent role in the clinical presentation. Consequently, patients may erroneously have been diagnosed as experiencing panic attacks for an extended period, leading to therapeutic failures [4]. In this report, we describe the electro-clinical features of a young female patient with focal epilepsy misdiagnosed for 15 years as PAs, aiming to highlight the crucial role of video–EEG monitoring in the differential diagnosis.

## 2. Materials and Methods

The present case report has been redacted according to the Declaration of Helsinki, the Guidelines for Good Clinical Practice, and the Strengthening the Reporting of Observational Studies in Epidemiology (STROBE) guidelines [5], and it has been approved by the local ethics committee for human experimentation.

### 2.1. Neuropsychological Evaluation

Neuropsychological assessment is performed during hospitalization. Intelligence quotient (IQ) and cognitive screening were assessed by using the Wechsler Adult Intelligence Scale—Fourth Edition (WAIS-IV) [6] and EpiTrack [7]. Manual dominance was evaluated by using the Oldfield Scale (OS) [8]. Attention and executive functions were assessed by Frontal Assessment Battery (FAB) [9], Stroop Test Time Effect (ST-TE) and Error Effect (ST-EE) [10], Trail Making Test (TMT) parts A and B [11], Digit Span Forward and Backward (DS-F, DS-B) [12], Babcock Story Recall Test (BSRT) [13], and Rey–Osterrieth complex figure—Delayed Copy (ROcf-DC) [14]. Visuo-spatial organization and perception were evaluated by using the Clock Drawing Test (CDT) [15] and Visual Discrimination of Segments (VDS) [16]. Breadth and organization of the lexical store were evaluated by using Verbal Fluency for letter (VF-L) and for category (VF-C) [17]. Praxis aspects were assessed by using the Rey–Osterrieth complex figure—Immediate Copy (ROcf-IC) [14]. The presence/absence of psycho-behavioral symptoms was evaluated with the Symptoms Checklist-90-R (SCL-90-R) [18].

### 2.2. Video–EEG/Polygraphic Monitoring

In cases where clinic and standard EEG are not sufficient to define a differential diagnosis between epileptic and non-epileptic seizures, it is necessary to document the critical event using long-term video–EEG monitoring (LTVEM). The method consists of the simultaneous video and EEG (VEEG) recording of the patient 24 h a day, for a duration varying from approximately 1 to 15 days, allowing for the acquisition of clinical data together with neurophysiological data. The detailed study of the electro-clinical characteristics of seizures recorded by VEEG monitoring has multiple indications and, therefore, is currently used for the following:

- Differential diagnostics between epileptic and non-epileptic seizures;
- Identification of the type of epileptic seizures and their frequency;
- Pre-surgical evaluation in patients with drug-resistant epilepsy.

In particular, in the context of pre-surgical diagnoses of drug-resistant epilepsies, LTVEM constitutes the gold standard test [19,20,21,22]. In our study, the parameters of the video–EEG/polygraphic recordings included video–EEG (electrodes were placed based on the 10–20 International System with bipolar montage; we also used additional zygomatic electrodes), EKG, and thoracic respiration (monitored using a strain gauge). Signals were acquired digitally (sampling frequency: 512 Hz; band-pass filters: 1.6–210 Hz; Nihon Kohden, Tokyo, Japan).

## 3. Case Report

### 3.1. Patient Information

The present report describes the case of a 25-year-old right-handed female with 16 years of formal education. She was born full-term via planned caesarean section with a nuchal cord, but no clear perinatal respiratory distress was reported. Beginning at the age of eight, the patient started experiencing paroxysmal episodes marked by a sudden sensation of fear, accompanied by palpitations and nausea. These episodes typically lasted about one minute and were followed by rapid recovery. Initially diagnosed as panic attacks, the symptoms were non-responsive to treatment with citalopram at a dosage of 20 mg SID.

Upon her admission to our research hospital, the patient underwent an extensive series of electro-clinical, neuropsychological, and neuroimaging evaluations aimed at accurately diagnosing the nature of her episodes.

### 3.2. Clinical Findings

Neurological examination showed normal function across various assessments including sensorium, cranial nerves, motor skills, sensory perception, cerebellar function, gait, reflexes, signs of meningeal irritation, and long-tract signs. She reported a noticeable decline in memory, particularly worsening over the past two weeks. Comprehensive blood tests were conducted, including complete blood count, assessments of kidney and liver function, serum lipid levels, glucose, lactate, lactic acid dehydrogenase, serum immunoglobulins, and thyroid hormones, and a panel of autoantibodies (ANA, ENA, ANCA, APL, anti-TPO), and returned normal results.

As per the neuropsychological assessment, on the WAIS-IV, a total score of IQ = 92 was reported; this score falls within the “average” range of cognitive functioning when compared to the reference population, as detailed in Table 1. A more comprehensive cognitive assessment revealed a mild impairment in visuo-spatial skills. All other cognitive functions examined were normal: executive functions, short- and long-term memory, working memory, divided, selective, and sustained attention, praxis skills, and ability to access the lexical store on phonemic and semantic stimuli (Table 2). No psycho-behavioral symptoms were reported (Table 3).

**Figure 1:**
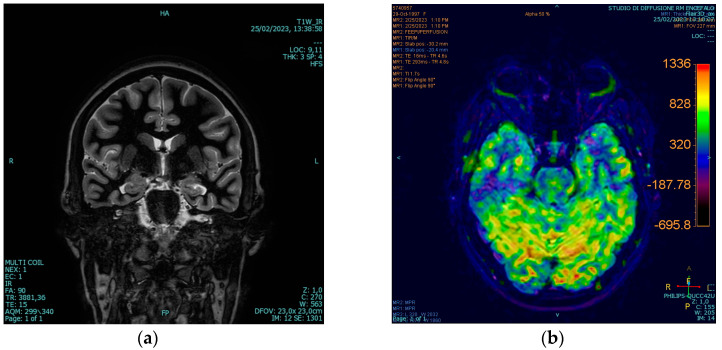
Three-Tesla MRI images and diffusion-weighted imaging (DWI) acquired. (**a**) Dysplastic lesion in right amygdala; (**b**) perfusion-weighted sequences.

In the context of the neuroimaging study, the patient underwent a 3-Tesla MRI of the brain. This imaging revealed a potential dysplastic lesion in the right amygdala (Figure 1a), associated with local hyperperfusion in perfusion-weighted sequences (Figure 1b).

### 3.3. Timeline and LTVEM

LTVEM was performed from 4.12 p.m. on 20 February 2023 to 11.30 a.m. on 20 February 2023. It documented five relevant epileptic seizures in wakefulness, clinically characterized by the following:

Episode 1 (at 3.43 p.m. on 20 February 2023): The patient saw a vision of “images” associated with anxiety and intense fear, with a tendency to bring her right hand to her mouth and for oromotor automatisms, including vomiting. The patient sought help from medical–nursing staff and called her parents with her phone for assistance.

Episode 2 (at 11:11 a.m. on 20 February 2023): The patient showed an altered perception of environmental brightness and intense fear, with a tendency to bring her right hand to her mouth and for oromotor automatisms, including chewing. The patient talked, sought help from medical–nursing staff, and could comprehend and follow simple commands.

Episode 3 (at 3:24 p.m. on 20 February 2023): The patient had a sensation of nausea and altered perception of environmental brightness, with intense fear (called her father on the phone for help) and a tendency to bring her right hand to her mouth, with oromotor, chewing, and head exploratory automatisms. The patient talked, answered simple questions, and sought help from medical–nursing staff.

Episode 4 (at 7:40 a.m. on 20 February 2023): The patient had intense fear with a tendency to bring her right hand to her mouth and for oromotor automatisms, including chewing. The patient was able to talk.

Episode 5 (at 11:20 a.m. on 20 February 2023): The patient had intense fear associated with visual hallucinations (reports of seeing people in the room watching her and telling her to breathe), a tendency to bring her right hand to her mouth, and oromotor automatisms, including chewing. The patient talked and sought help from medical–nursing staff.

**Figure 2:**
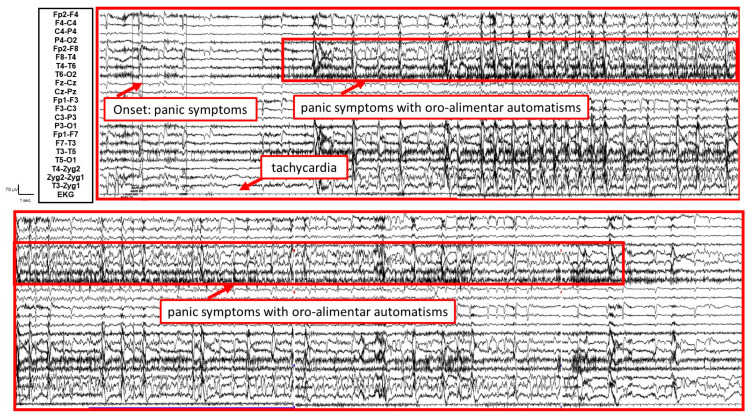
Video–EEG/polygraphic findings of a seizure. A rhythmic sharp and slow theta activity predominantly over the right temporal (red) and zygomatic derivations, in part obscured by muscular chewing and swallowing artifacts, was observed, associated with tachycardia.

From an electroencephalographic perspective, a rhythmic sharp and slow theta activity predominantly over the right temporal and zygomatic derivations, in part obscured by muscular chewing and swallowing artifacts, was observed, associated with tachycardia (Figure 2).

### 3.4. Diagnosis

LTVEM successfully documented five epileptic seizures. Clinically, these episodes were primarily characterized by an overwhelming sensation of fear, nausea, and visual hallucinations. Correspondingly, the EEG recordings indicated paroxysmal activity predominantly in the right temporal and zygomatic leads. Notably, during these episodes, the patient, who is right-handed, was able to speak and alert others. Additionally, magnetic resonance imaging of the brain confirmed a potential dysplastic lesion in the right amygdala.

Given the clinical presentations and the corroborative diagnostic findings, the patient was diagnosed with focal seizures, rather than panic attacks (PAs). She is a candidate for surgical treatment due to her drug-resistant focal epilepsy.

## 4. Discussion

PAs and IF are two distinct nosological entities that exhibit different clinical characteristics, upon which the differential diagnosis can typically be based. In particular, the differences pertain to the state of consciousness (in IF, the patient may not be alert, while in PAs the patient is always alert), the duration (in IF, the episode lasts between 0.5 and 2 min, while in PAs, it lasts between 5 and 10 min), the content of the thought (in IF, dissociative phenomena may be present such as amnesia of the event, hallucinations, or sensations of déjà vu, with a prevalence > 5%, while in PAs, the aforementioned phenomena are very rare), automatisms (in IF, they are common, with progression toward complex partial seizures, while in PAs, they are infrequent), agoraphobia (in IF, it is not present, while it is present in PAs), depressive symptoms (in IF, they may be present but are not associated with the severity of the seizures, while in PAs, they are common and associated with the severity of the events), and anticipatory anxiety (in IF, it is not common, while in PAs, it is very frequent). Regarding the age of onset, PD typically manifests during late adolescence or early adulthood [4]. Ictal fear is generally characterized by a lack of response to common psychiatric therapies used in the treatment of PD [23], although benzodiazepines (e.g., clonazepam) are often employed for this disorder, benefiting both epileptic and non-epileptic patients [24]. Concerning the patient’s medical history, factors such as perinatal hypoxic distress or a history of head trauma, which can predispose them to the development of epilepsy, can aid in the differential diagnosis between partial epilepsy and panic disorder [25].

However, if a differential diagnosis is not clear, especially without an ictal VEEG recording, the overlapping of symptoms between PD and IF may represent a complex diagnostic challenge. Both of these nosological entities in fact share other potential clinical manifestations besides fear, especially autonomic symptoms such as tachycardia, tachypnea, and/or dyspnea and fluctuations in blood pressure [3]. Furthermore, panic is the anxiety disorder most frequently associated with seizures in the so-called “ictal anxiety” [26], and this can make the differential diagnosis even more challenging. People with epilepsy tend to have panic attacks approximately six times more frequently than control populations, with a point prevalence of 15–30% [27,28,29,30].

The epileptogenic focus of ictal fear is usually located within the mesial structures of the right temporal lobe, with the amygdala playing a pivotal role [4]. The role of the amygdala in the genesis of ictal fear has been demonstrated through deep-brain electrical stimulation studies during pre-operative evaluations of patients referred for surgical treatment of epilepsy, as well as by MRI findings showing volumetric atrophic alterations in the amygdala in patients with ictal fear [31]. Furthermore, it is believed that within the “ictal fear circuit”, frontal lobe structures such as the anterior cingulate cortex and orbitofrontal cortex are also involved following the initial discharge from the temporal lobe [32]. It is worth noting that manifestations accompanying ictal fear, such as orobuccal automatisms, the presence of olfactory aura/hallucinations, disperceptive phenomena like visual and auditory hallucinations, and visceral sensations, all suggest a genesis in the anteromesial structures of the temporal lobe [33].

Our electro-clinical, neuropsychological, and neuroimaging data strongly indicate and support the presence of a right temporal epileptogenic focus. A review of focal epilepsy with ictal fear highlighted that structural alterations in the right hemisphere were found in 86% of cases, particularly affecting the temporal lobe structures [4]. Regarding the lateralization of the genesis of ictal fear, while acknowledging that most research data point to right hemisphere localization, consistent with the findings presented in this case report, it is noteworthy that Straus et al. reported a non-preferential lateralization of the epileptogenic focus in the patients they studied, suggesting that basic components of fear may have a bilateral representation [34].

Temporal lobe involvement does not seem to be the exclusive epileptogenic focus for ictal fear. Alemayehu and colleagues described two cases of patients with structural focal parietal lobe epilepsy, where the cardinal symptom was a sudden sensation of panic. In both cases, recordings from combined depth and subdural grid arrays revealed that the ictal symptomatology was solely dependent on the epileptogenic activity of the parietal focus, excluding potential spread to mesiotemporal structures [24].

Finally, our report emphasizes the role of VLVEG in differentiating focal epilepsy from PAs, leading to a correct and appropriate diagnosis in order to avoid many years of misdiagnosis, treatment inefficacy, and inappropriate management.

## Figures and Tables

**Table 1:** Wechsler Adult Intelligence Scale—Fourth Edition (WAIS-IV) scores.

	Sum of Weighted Scores	Composite Score	Percentile Rank
VCI (Verbal Comprehension Index)	28	96	39
PRI (Perceptual Reasoning Index)	28	98	43
WMI (Working Memory Index)	17	92	29
PSI (Processing Speed Index)	15	86	18
**TIQ (Total Intelligence Quotient)**	**89**	**92**	**28**

**Table 2:** Cognitive assessment scores.

TEST	Score	Outcome
OS	12	Right-handed
**Screening**		
EpiTrack	36	In the norm
**Attention and executive functions**		
FAB	18	In the norm
ST-TE	15	In the norm
ST-EE	0	In the norm
TMT—A	40	In the norm
TMT—B	85	In the norm
TMT—B-A	44	In the norm
**Memory**		
DS-F	4.5	To the limit of the norm
DS-B	4	To the limit of the norm
BSRT	15.5	In the norm
ROcf-DC	14.25	In the norm
**Visuo-spatial organization and perception**		
CDT	3	Mild impairment
DVS	26	In the norm
**Breadth and organization of the lexical store**		
VF-L	40	In the norm
VF-C	70	In the norm
**Praxis**		
ROcf-IC	31.25	In the norm

**Table 3:** Symptoms Checklist-90-R (SCL-90-R) scores.

	Score	Cut-Off
Global Score Index (GSI)	0.18	<0.566
Somatization (SOM)	0.75	<1.50
Obsession–compulsion (O-C)	0.20	<1.50
Interpersonal sensitivity (INT)	0.00	<1.50
Depression (DEP)	0.08	<1.50
Anxiety (ANX)	0.20	<1.50
Hostility (HOS)	0.00	<1.50
Phobic anxiety (PHOB)	0.00	<1.50
Paranoid ideation (PAR)	0.00	<1.50
Psychoticism (PSY)	0.00	<1.50
Sleep disorders (SLEEP)	0.33	<1.50

## Data Availability

The data presented in this study are available on request from the corresponding author. The data are not publicly available due to patient privacy concerns.
